# Glycemic Responses, Appetite Ratings and Gastrointestinal Hormone Responses of Most Common Breads Consumed in Spain. A Randomized Control Trial in Healthy Humans

**DOI:** 10.3390/nu7064033

**Published:** 2015-05-27

**Authors:** Carolina Gonzalez-Anton, Maria C. Rico, Estefania Sanchez-Rodriguez, Maria D. Ruiz-Lopez, Angel Gil, Maria D. Mesa

**Affiliations:** 1 Department of Biochemistry and Molecular Biology II, Institute of Nutrition and Food Technology “Jose Mataix”, University of Granada, Avenida del Conocimiento s/n, 18100 Armilla (Granada), Spain; E-Mails: karol29@correo.ugr.es (C.G.-A.); mdcrico@ugr.es (M.C.R.); estefaniasr@outlook.com (E.S.-R.); mdmesa@ugr.es (M.D.M.); 2 Department of Nutrition and Food Science, Institute of Nutrition and Food Technology “Jose Mataix”, University of Granada, Campus Universitario de Cartuja C.P., 18071 Granada (Granada), Spain; E-Mail: mdruiz@ugr.es

**Keywords:** appetite ratings, bread, dietary fiber, gastrointestinal hormones, glycemic index, glycemic load, insulinogenic index

## Abstract

The present study was carried out to determine the glycemic index (GI), glycemic load (GL), insulinemic index (InI), appetite ratings and postprandial plasma concentrations of gastrointestinal hormones related to the control of food intake after the ingestion of the five most common breads consumed in Spain with different compositions and manufacturing processes. Twenty-two healthy adults participated in a randomized crossover study. The breads tested were Ordinary, Precooked-Frozen, Candeal-flour, Alfacar whites and Wholemeal. All breads portions were calculated to supply 50 g of available carbohydrates. In addition, 50 g of glucose was used as a reference. A linear mixed-effects model was used to compare data calculated for all breads with glucose load. The GI value varied from 61 for the Wholemeal, to Alfacar 68, Ordinary 76, and 78 and 86 for the Precooked-Frozen and Candeal-flour breads, respectively. Wholemeal and Alfacar had lower GI than glucose. All tested breads had a lower GL (ranged 9 to 18) compared with glucose. Wholemeal GL was similar to Alfacar, but lower than the other white breads. InI were significantly lower for all breads (ranged 68 to 73) compared with glucose, and similar among them. The intake of the Wholemeal bread led to a higher release of gastric inhibitory polypeptide compared with the Ordinary and Precooked breads and to a higher release of pancreatic polypeptide compared with the Precooked-Frozen bread. All breads affected appetite ratings similarly. In conclusion, based on GL, the Wholemeal bread would be expected to exert a favorable glycemic response.

## 1. Introduction

Mediterranean diet is characterized by a high consumption of cereals, primarily as whole grains, and is associated with a lower incidence of mortality from all causes [1]. It is necessary to consume four to six servings of cereals and derivatives to achieve carbohydrate intake recommendations; indeed, if half of this amount comes from whole-grain products, meeting fiber requirements are guaranteed [2]. However, consumption of cereals and derivatives has experienced a marked decrease in recent decades, especially in developed countries [3]. The most important cereal-derived nourishment is bread, a staple food that is the main source of daily carbohydrates. The most rapid decline has occurred for white bread and is most likely because of a popular belief that bread provokes weight gain. Specifically in Spain, bread consumption has declined from 368 g/d in 1964 to 139 g/d in 2012 [4]. However, the overall obesity prevalence in Spain has dramatically increased by 27% over the last two decades [5]. 

Glycemic index (GI) is a source of controversy. Two recent meta-analyses concluded a positive association between both GI and GL with the incidence of type 2 diabetes [6,7] and heart diseases [7], and found consistent evidences related to the protective effects of low dietary GI and GL [6]. However, low GI, low-carbohydrate diet, compared with the high-GI, high-carbohydrate diet, did not affect insulin sensitivity, systolic blood pressure, LDL cholesterol, or HDL cholesterol but lowered triglycerides [8]. In fact, these authors reported that at a high dietary carbohydrate content, low- compared to high-GI significantly reduced insulin sensitivity, while at a low carbohydrate content, low- compared to high-GI did not affect insulin sensitivity but increased fasting blood glucose levels [8]. In addition, related to obesity, no associations among body mass index (BMI), GI or GL have been reported in a Mediterranean population [9]. Thus, investigations focused on the implication of glycemic response can be useful in order to determine the utility of using GI to select specific foods that may improve cardiovascular risk factors or insulin resistance. Indeed, the recent criticisms of the GI focus on its validity and stated that GI methodology is not well standardized and has several flaws [10], and that GI values do not represent a valid property of foods to predict which foods are healthy [11]. However, Wolever [12] has justified the validity since: (1) GI methodology is accurate and precise enough for practical use; (2) GI is a property of foods; and (3) GI is biologically meaningful and relevant to virtually everyone [12]. Besides, GI, GL and insulinemic index (InI) help the evaluation of glycemic response and the effect of carbohydrates on health [13,14]. Therefore, the determination of GI, GL and InI can give a complete view of the glycemic metabolic response. 

Many factors, such as manufacturing conditions, starch structure, bread particle size and inclusion of different types of ingredients such as whole-grains, may influence the glycemic response and therefore may contribute to modify the GI, GL and InI of breads [15,16,17,18,19]. Comparing different French breads, the GI value varied between 57 for the traditional baguette and 85 for the wholemeal loaf, whereas the traditional baguette exhibited the significantly lowest postprandial insulin response and the lowest InI [16]. These authors justify that the glycemic response of breads may be influenced by ingredients and manufacturing conditions (fermentation, gelatinization, organic acid generation), which leads to differences in physical characteristics [17]. Hence, the aim of the present study was to evaluate the GI, GL and InI of five different most common breads consumed in Spain with different compositions, physical characteristics and manufacturing processes. As secondary outcomes we have determined the influence of these breads on postprandial plasma concentrations of gastrointestinal hormones involved in the control of satiety and hunger feelings, as well as on appetite ratings and palatability in healthy adults.

## 2. Participants and Methods

### 2.1. Study Design

A prospective, crossover, randomized, single-blind controlled study was carried out to evaluate five different types of bread. Breads were selected from the most common and frequently consumed in Spain: Ordinary white, Precooked-frozen white, Candeal-flour white, Alfacar white and Wholemeal breads. The nutritional composition of breads is specified in Table 1. Every morning, a baker provided freshly baked breads made according to standardized ingredients and manufacturing conditions as specified below. The amount of bread was weighted to supply 50 g of available carbohydrates (CHO) for each volunteer (Table 1). The portion sizes given to the subjects are indicated in Table 1.

**Table 1:** Composition of the different tested breads.

	Energy (kcal/100 g)	Protein (%)	Fat (%)	CHO (%)	Fibre (%)	Ash (%)	Moisture (%)	AV-CHO (g)	AV-CHO per Serving (40 g) ^γ^	Bread Provided (g (kcal)) *
**Ordinary**	283	9.3	1.3	56.5	4.1	2.3	30	52.4	21.0	95 (270)
**Precooked-Frozen**	271	8.8	1.2	54.2	4.3	2	33.7	49.9	20.0	100 (270)
**Candeal-flour**	275	7.6	1.3	56.6	3.3	2.2	32.2	53.3	21.3	94 (258)
**Alfacar**	282	9.3	1.3	56.6	3.5	2	30.5	53.1	21.2	94 (266)
**Wholemeal**	243	8.8	1.2	45.3	7.9	1.8	41.6	37.4	15.0	134 (325)

* Amount of bread (g) providing 50 g of available carbohydrate and its corresponding energy value. **^γ^** Available carbohydrates content in 40 g per serving of bread. CHO, carbohydrates; AV-CHO, available carbohydrates.

This study was conducted according to the guidelines set in the Declaration of Helsinki and was approved by the Ethics Committee of the University of Granada. All subjects gave written informed consent to participate in the study. This trial was registered at clinicaltrials.gov as NCT02297074.

### 2.2. Subject Selection

A total of 23 healthy adults (13 males and 10 females) from 19 to 32 years old (mean age: 25 ± 1 years) with a body mass index (BMI) ranging from 19.2 to 28.5 (mean BMI: 23.3 ± 0.5) (17 subjects with normal weight and 5 moderately overweight) and used to eating bread were initially selected from December 2012 to January 2013 from a group of Human Nutrition and Dietetics Degree students at the University of Granada. Exclusion criteria were: less than 18 years old or more than 45 years old, BMI lower than 18 or more than 29, smoker, pregnant or breastfeeding, unusual fiber consumption, glucose plasma levels higher than 110 mg/dL, insulin plasma levels higher than 10 mU/mL, blood pressure higher than 110 mmHg, medication to treat blood pressure, glucose or lipid metabolism, suffering from metabolic or gastrointestinal syndromes, genetic dyslipidemia, intake of nutritional supplements in the last three months, on a diet in the last three months or practicing endurance sports. To avoid menstrual cycle disturbances, women on the menstruation days will be rescheduled for the following week.

To avoid the risk of reaching false conclusions, psychometric validations of food restrictions were determined for all subjects using the revised version of the Three Factor Eating Questionnaire (TFEQ) [20]. The 21-item TFEQ is a composite score with a scale that measures 3 domains of eating behavior: cognitive restraint, uncontrolled eating, and emotional eating. This measure has been reported to be a useful tool for characterizing these 3 domains, showing robust factor structure and good reliability [20]. Those with a TFEQ score lower than 2.46 were selected (*n =* 23). 

The sequence of bread intake for each subject was selected by a random system. All subjects started taking one randomly selected bread and continued according to the selected sequence. Fifty grams of control glucose dissolved in 200 mL water was administered in the same conditions as breads on two separate occasions, one before and one after intake of the five breads, as recommended by Brouns *et al.* [21]. The intake of each bread or glucose was separated by at least one week (wash-out period).

Analyses were blinded to analysts. A complete clinical history of each subject, including demographic data (age, sex, origin and family history) and disease background, as well as the current use of any drugs were registered. One subject was excluded from the study because some problems occurred during blood extraction, leaving a total of 22 subjects that were included in the final design. 

### 2.3. Assessed Breads

The Ordinary white bread was made with wheat flour, water, yeast, salt, additives and 9% sourdough. All ingredients were placed together in the recommended order, mixed and the dough was kneaded (at 24 °C) 4 min at low speed and 8 min at high speed until well developed. The dough rested for 25 min and then it was gently degassed by pressing the dough out. Stick loaves were shaped and were left for fermentation in a warm, moist environment (75% humidity) at 29 °C for 90 min. During this time, the loaf rose until it almost doubled in size. After this period of time, loaves were placed in a hot preheated oven at 210 °C until golden and baked through (30 min). Then, baked loaves were immediately turned onto a wire rack to cool. The resulting bread is characterized by a white crumb with regular soft alveoli and a slightly soft thin crust.

The Precooked-Frozen bread was baked in two steps as the unique difference compared with the Ordinary bread; the fermentation procedure and baking process were similar. A first cook (180 °C, 16 min) was carried out before cooling at 30 °C for 18 min and then freezing at −30 °C for 30 min. Then loaves were packed and preserved at −18 °C until use. After defrosting, the second cook was done at 230 °C for 12 min. The resulting bread is characterized by a white crumb with regular soft alveoli and a bright crusty crust.

The Candeal-flour white bread is a compact and dense loaf of bread of hard dough made from Candeal-wheat flour, which is poor in gluten; it contains only 11% protein. Candeal-flour bread has compact dough, as it has no resting period after mixing the basic ingredients to avoid premature fermentation and, thus, generation of organic acids. This type of bread was made with Candeal-wheat flour, water, yeast, salt, additives and 20% sourdough; ingredients were mixed and the dough was kneaded (at room temperature) until well developed (3–5 min). The kneading process finishes in a refiner cylinder that provides a special texture to the bread. Immediately, the dough was left for a first block fermentation. Stick loaves were degassed, shaped and were left for fermentation at room temperature for 90 min. After this period of time, loaves were placed in a hot, moist, preheated oven at 210 °C for 30–35 min. Then, baked loaves were immediately turned onto a wire rack to cool. The resulting bread is characterized by a thick crust between one and two milimeters thick, which is smooth and crisp, golden to light brown in color and tastes of toasted cereal. The crumb of the bread is white and its texture is smooth, spongy and consistent, with little regular alveoli (looks like cotton) and an intense cereal aroma with a pleasant and slightly sweet taste. 

Alfacar white bread was made by hand for at least one stage of the bread-making process, according to its protected geographical indication (I.G.P. Alfacar, Granada, Spain). All ingredients (low baking capacity wheat flour, Alfacar low-salt spring water, yeast and edible salt) are obtained in the Alfacar area. The dough is made in 21 min at 27 °C using approximately 25% leavened dough. The dough block rests from 5 to 20 min and then is divided into a unique shape with stick leaves (15 min). Fermentation is carried out on wooden planks (1–2 h) and then the dough was cooked at 200–230 °C for 25–30 min. Thus, the manufactured bread has a creamy white, flexible and soft crumb, with many randomly scattered holes that vary in size. The crust is medium-thick to thick, golden, slightly shiny and quite smooth. It has a characteristic aroma of fermented vinegar and/or milk that is produced in dough fermentation, which may be mild to relatively strong. 

The Wholemeal bread was made from wholemeal wheat flour (with 80% of extraction), water, yeast, salt and 40% sourdough. All ingredients were placed together in the recommended order, mixed and the dough was mechanically kneaded (at room temperature) until well developed (10 min); salt was added at the end of the kneading process. The dough rested for 2 h at room temperature and then it was degassed, divided into smaller pieces that rested for 30 min. Loaves were shaped and were left for fermentation in a warm, moist environment, at room temperature for 120 min. After this period of time, the surface was cut and loaves were placed in a hot preheated oven at 200 °C for 40 min. Then, baked loaves were immediately turned onto a wire rack to cool. The resulting dark brown bread was characterized by a compact crumb free of alveoli and a hard thin crust.

### 2.4. Study Performance

The evening before the test day, the subjects consumed a standardized dinner supplied by the study team. The meal consisted of pizza and pineapple juice (800 kcal: protein 18% of energy (E), fat 22%E and carbohydrate 60%E). The subjects were instructed to always have dinner at the same time, before 10:00 p.m. and to not eat or drink anything other than half a liter of water after dinner. The subjects were instructed to refrain from alcohol and/or from performing intense physical activities 48 h prior to each test day.

Volunteers arrived at 8 a.m. in a fasting state at the Institute of Nutrition and Food Technology “José Mataix” of the University of Granada (Spain) by car or bus. After resting 20 min, anthropometric measurements (weight, height and waist circumference) were determined the first day of intervention by the same member of the professional staff. For all measurements, participants were without shoes and wore indoor clothing. BMI was calculated as weight (kg) divided by height squared (m^2^). Bioimpedance analysis was used to estimate body composition (fat, lean, fat free and bone body mass and total water) based on differences of tissues conductivity, by using a TANITA BC-420MA. On each day of the study, a fasting blood sample was acquired after the resting time, and appetite feelings were assessed by using visual analogue scales (VAS) (as described below) [22].

The subjects were instructed to consume the tested bread with 150 mL of water within 12 min. The subjects immediately completed two VASs: one on appetite feelings and another on bread palatability. Volunteers completed a VAS to evaluate sensory acceptance: appearance, smell, taste, and palatability. The appetite feeling VASs were repeated every 30 min until a total of 180 min had passed. On the first and last day of the intervention, volunteers consumed 50 g of glucose dissolved in 200 mL. The subjects were not allowed to eat or drink anything else during the 180 min of the intervention. They were allowed to read, study, talk or listen to quiet music, but they were not permitted to sleep.

At fasting state, a catheter was placed in the antecubital vein, and a baseline blood sample was collected. After collection of the first blood sample (time 0), subjects ingested the bread within 10 min. Thereafter, blood samples were taken at time 15, 30, 45, 60, 90, 120 and 180 min following the intake of the breads or glucose. After the last blood extraction (3 h), an *ad libitum* lunch consisting of a Bolognese macaroni (protein 16%E, fat 27%E and carbohydrate 57%E) and 300 mL of water was provided. Volunteers ate *ad libitum* until comfortably satisfied and the amount of food intake was registered by differences in pasta weight before and after lunch; the *ad libitum* energy intake was calculated afterwards. After the *ad libitum* lunch, the subjects completed appetite ratings on VASs. The participants completed a 48-h dietary survey diary, which included the food intake from the day before and the day of the intervention. 

## 3. Methodology

### 3.1. Analytical Methods

Blood was collected into EDTA-coated tubes. Immediately after the blood sample was obtained, the blood was centrifuged at 1000*× g* for 15 min at 4 °C and plasma was divided into aliquots and frozen at −80 °C for glucose analysis, which was determined spectrophotometrically. For the determination of insulin and gut hormone plasma concentrations, whole blood was added to Pefabloc SC (AEBSF) (Roche), which is needed for ghrelin determination (1 mg/mL), and dipeptidyldipeptidase IV inhibitor (Linco), which is needed for the determination of glucagon-like peptide-1 (GLP-1) (50 μM). This blood sample was centrifuged at 1000*× g* for 10 min at 4 °C, divided into aliquots and frozen at −80 °C for analyses. Plasma concentrations of insulin, ghrelin, GLP-1, gastric inhibitory polypeptide (GIP), peptide YY (PYY) and pancreatic polypeptide (PP) were determined using a MILLI*plex™* kit, with the Luminex 200 multiplex assay system built on xMAP technology with the Human Gut Hormone Panel (Millipore Iberica S.A., Madrid, Spain) as previously we described [23]. 

The areas under the curves (AUCs) of postprandial glucose, insulin, gastrointestinal hormone, as well as for VAS time-courses were calculated using a trapezoidal method with R statistical software [24]. 

### 3.2. GI, GL and InI Calculations

Our primary outcomes were to determine GI, GL and InI. GI is defined as the blood glucose response from time 0 until 120 min, measured as the AUC in response to a test food consumed by an individual under standard conditions related to the postprandial response to 50 g of oral glucose load under the same conditions [21]. The test food and reference food (50 g glucose) must contain the same amount of available carbohydrates. GL takes into account the GI of the product and how many available carbohydrates are in a food serving and is calculated as GL = (GI of product/100*×* g of available carbohydrates in a food serving). According to FINUT healthy lifestyles guide [25] we considered that each bread serving consisted of 40 g. InI measures the increment of insulin AUC over 120 min in response to consumption of the amount of bread providing 50 g of available carbohydrates divided by the AUC after ingestion of 50 g of glucose after two hours [26,27]. 

### 3.3. Appetite Profile Determination 

Appetite profiles were assessed using VAS ratings of hunger, satiety, fullness and prospective food consumption, based on a 100-mm scale ranging from 0 (“not at all”) to 100 (“extremely”) [28]. This questionnaire was completed before breakfast intake and every 30 min for 180 min following breakfast intake. A validated composite appetite score was calculated using the following equation: composite appetite score = (satiety + fullness + (l00 − prospective food consumption) + (100 − hunger))/4 [28]. Additionally, information regarding the appearance and palatability of the breakfasts and lunches was also recorded. Sensory acceptance was assessed using VAS ratings of appearance, smell, taste, and palatability, based on a 100-mm scale ranging from 0 (“very good”) to 100 (“very bad”) [28]. This questionnaire was completed after breakfast to register differences among the breads.

### 3.4. Statistical Analysis

In accordance with Brouns *et al.* [21], the minimum sample size to determine GI is 10 subjects. In addition, for a longitudinal study, with 6 sequences or periods of measurements, for a medium size effect (0.3) and assuming a variance between repeated measure of 0.5 and power of 0.9, the estimated sample size needed is 19 independent participants. Including a potential 10% dropout rate, the minimum sample size is 21 participants. Values are presented as the mean ± SEM. A linear mixed-effects model (LMM) adjusted by age and gender and using Sidak test for the marginal means was used to compare GI, GL, InI, *ad libitum* energy intake, appetite scores, sensory acceptance and gastrointestinal hormones AUCs calculated for all breads and glucose load as well as absolute values at each time point. This method of analysis repeats measures over time and considers the correlation of responses within subjects. The fixed effects chosen were treatment, age, gender and BMI; these effects were analyzed and eliminated if there were no significant changes. Association for global relations between GI, GL, InI, *ad libitum* energy intake, appetite scores and gastrointestinal hormones AUCs after consuming all breads, were calculated by LMM. Coefficients of association were obtained from data after consuming the five breads and glucose. A *p* value of <0.05 was considered significant. SPSS (Statistical Package for the Social Sciences) version 20 software was used to perform the statistical analysis (SPSS Inc, Chicago, IL, USA). 

## 4. Results

### 4.1. Baseline Subject Characteristics

Twenty-two participants completed the seven test days according to the protocol. Table 2 summarizes the subjects’ baseline characteristics. The mean BMI, waist circumference, body fat, lean body mass, fat free body mass, total water, bone mass and basal metabolism (kcal) were within normal ranges. Five of twenty-two selected volunteers were healthy overweighed (3 M/2 F).

**Table 2:** Baseline demographic and anthropometric characteristics of the volunteers.

	Mean ± SEM
Gender (Male/Female)	(12/10)
Age (years)	26 ± 1
BMI (kg/m^2^)	23.8 ± 0.5
Waist circumference (cm)	76 ± 2
Body fat (kg)	15.2 ± 1.2
Lean body mass (kg)	53.1 ± 2.2
Fat free body mass (kg)	50.4 ± 2.1
Total water (kg)	37.2 ± 1.5
Bone mass (kg)	2.7 ± 0.1
Basal metabolic rate (kcal)	1594 ± 59
Values are expressed as the mean ± SEM (*n =* 22).	

BMI, body mass index.

### 4.2. GI, GL and InI

The GI values were 61 for the Wholemeal, 68 for the Alfacar, 76 for the Ordinary, 78 for the Precooked-Frozen and 86 for the Candeal-flour breads. There were no significant differences in GI among the different breads. However, the Wholemeal and Alfacar breads had lower GI than glucose (Table 3). All tested breads had lower GL (range 9 to 18) than glucose (all *p* < 0.001). Wholemeal bread had the lowest GL (9), similar to Alfacar (14) and lower than the rest of the breads (*p* = 0.024, *p* = 0.036 and *p* = 0.002 for the Ordinary, Precooked-Frozen and Candeal-flour breads, respectively). The InI ranged from 68 for the Ordinary and Precooked-Frozen to 73 for the Wholemeal; values were similar within breads but significantly lower for all breads compared with the glucose load (all *p <* 0.001) (Table 3). There were no differences in glucose and insulin AUCs among the five breads. 

**Table 3:** GI, GL and InI after the intake of the five breads.

	Ordinary	Precooked-Frozen	Candeal-Flour	Alfacar	Wholemeal
	Mean (CI 95%)	Mean (CI 95%)	Mean (CI 95%)	Mean (CI 95%)	Mean (CI 95%)
**GI**	76 (59/92)	78(54/89)	86 (70/103)	68 * (51/83)	61 * (44/76)
**GL**	16 ^b^* (12/18)	16 ^b^* (11/18)	18 ^b^* (15/22)	14 ^ab^* (11/18)	9 ^a^* (6/12)
**InI**	68 * (59–81)	68 * (57/78)	69 * (59/80)	70 * (60/81)	73 * (63/84)
	Mean ± SEM	Mean ± SEM	Mean ± SEM	Mean ± SEM	Mean ± SEM
**Glucose AUC (mg/dL·min)**	910 ± 125	854 ± 135	1133 ± 127	886 ± 125	828 ± 125
**Insulin AUC (mU/mL·min)**	2020 ± 247	1810 ± 254	1955 ± 248	1939 ± 248	2190 ± 248

Values are expressed as the mean ± SEM (*n =* 22). LMM was used to compare different breads and glucose adjusted by age and gender and using Sidak test for the marginal means. * Indicates differences *versus* glucose as reference (100 for GI and InI and 50 for GL). Different superscript letter indicates significant differences between breads. *p <* 0.05 was considered significant. AUC, area under the curve; CI, confidence interval; GI, glycemic index; GL, glycemic load; InI, insulinemic index; LMM: linear mixed-effects model; SEM: standard error of the mean.

### 4.3. Plasma Glucose and Insulin Postprandial Concentrations

**Figure 1:**
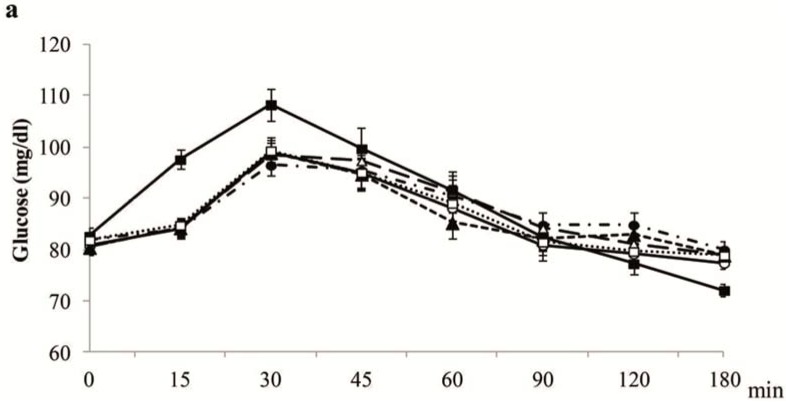

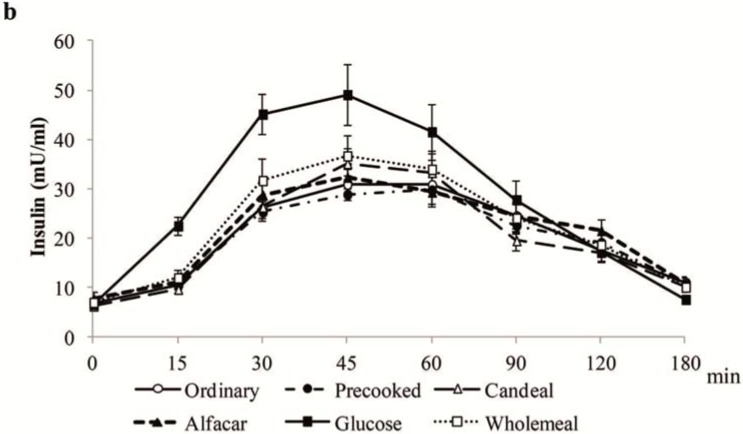
Plasma glucose (**a**) and insulin (**b**) concentrations. Values are expressed as the mean ± SEM (*n =* 20); SEM, standard error of the mean.

Figure 1a depicts the postprandial plasma glucose after the intake of the tested breads or glucose. Figure 1b shows the postprandial insulin curves after intake of the five tested breads and glucose solution. 

### 4.4. Postprandial Gastrointestinal Hormones Plasma Concentrations

**Figure 2:**
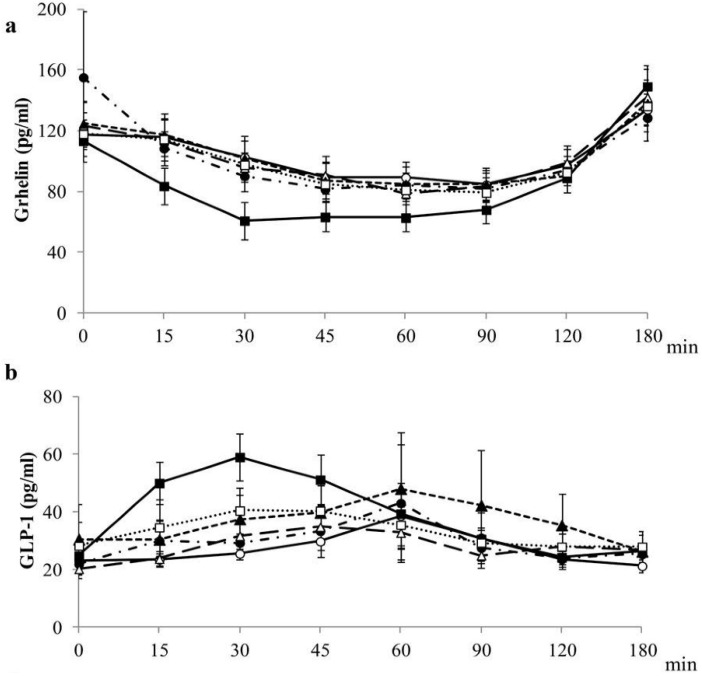

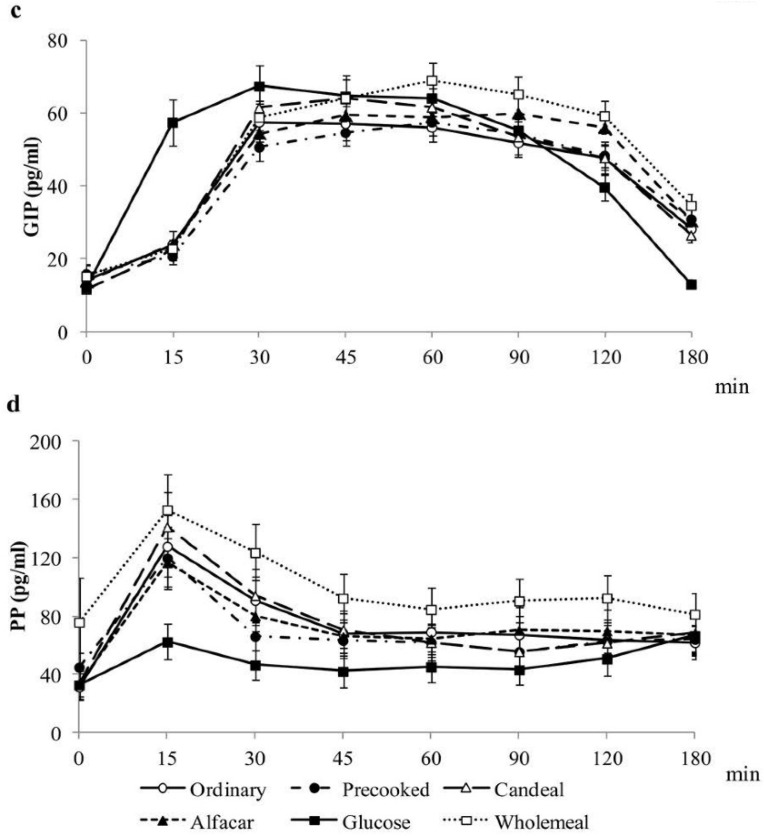
Plasma gastrointestinal hormones concentrations. (**a**) grhelin; (**b**) GLP-1; (**c**) GIP; (**d**) PP; values are expressed as the mean ± SEM (*n =* 20). GIP, gastric inhibitory polypeptide; GLP-1, glucagon-like peptide-1; LMM, linear mixed-effects model; PP, pancreatic polypeptide; SEM, standard error of the mean.

Postprandial plasma concentrations of gastrointestinal hormones involved in the control of appetite are shown in Figure 2 and Table 4. The release of GIP (AUC) was higher after the intake of the Wholemeal than after the Ordinary and Precooked-Frozen ingestion, while PP (AUC) was only higher after the intake of wholemeal than after the Precooked-Frozen ingestion; no differences were found from ghrelin, GLP-1 and PYY within breads (Supplemental Table S1). 

**Table 4:** Values of standardized coefficients of association for global relations between GI, GL and InI and *ad libitum* energy intake, appetite scores and gastrointestinal hormones AUCs after consuming the breads from all data obtained by LMM.

	GI	GL	InI
EI	0.013	0.116 **	0.127 *
Hunger neg	0.025	0.038	−0.034
Satiety	0.162 *	0.248	0.026
Fullness	0.183 *	0.297 *	0.069
Prospective consumption neg	0.066	0.086	0.039
CAS	0.118	0.18	0.017
Ghrelin neg	−0.069	0.059	−0.023
GLP-1	0.221 **	0.182 **	0.489 ***
GIP	−0.07	−0.029	0.151 *
PP	−0.111	−0.245 ***	−0.043
PYY	0.14	−0.017	0.347 ***

^1^ Coefficients of association for global relations were obtained from LMM from data after consuming all breads and glucose. Each volunteer consumed the five experimental breads and glucose; ^2^ Significant values: * *p* < 0.05, ** *p* < 0.01 and *** *p* < 0.001; ^3^ neg indicates a postprandial decrease of the parameter. AUC, area under the curve; CAS, composite appetite score; EI, energy intake; GI, glycemic index; GIP, gastric inhibitory polypeptide; GL, glycemic load; GLP-1, glucagon-like peptide-1; InI, insulinemic index; LMM: linear mixed-effects model; PP, pancreatic polypeptide; PYY, peptide YY.

### 4.5. Appetite Ratings and Sensory Acceptance

Appetite ratings, hunger, satiety fullness, prospective consumption and composite appetite score AUC were similar after the intake of all tested breads. In addition, desires to eat different types of flavors were also similar after the intake of all breads. All subjects ate a similar amount of calories 24 h before the intervention. No differences for *ad libitum* energy intake and in the calorie density eaten during the day of the intervention with the five types of breads were found (Supplemental Table S2). Bread appearance did not show any difference among the tested breads. Attending to taste and palatability ratings, no significant differences were obtained between white breads, while the Wholemeal bread had the lowest score. Finally, we observed that Ordinary bread and Candeal-flour bread had significantly higher scores for smell rating than Precooked-frozen bread and Alfacar bread, and that Wholemeal bread obtained the lowest score for smell (Supplemental Table S2).

### 4.6. Correlations between GI, GL, InI, Gastrointestinal Hormones Postprandial Concentrations, ad libitum Energy Intake and Appetite Scores

Table 4 shows the correlations between GI, GL and InI with *ad libitum* energy intake, appetite scores, and gastrointestinal hormones postprandial AUCs. We observed a significant positive relationship between GI and GL with satiety and fullness postprandial variations (AUCs). GL and InI were associated with *ad libitum* energy intake. Postprandial variation of GLP-1 was positively correlated with GI, GL and InI, the postprandial GIP and PYY AUCs with InI, while PP AUC was inversely related to GL (Table 4). No significant correlations between postprandial ghrelin, GLP-1, GIP and PP with appetite scores (AUCs) were found. Only PYY release was positively associated with the decrease of hunger (*r* = 0.282; *p* = 0.035). 

## 5. Discussion

In the present work, for the first time, we compared the GI, GL and InI of five most common breads commercially available in Spain with different compositions and manufacturing processes. Our hypothesis was that different ingredients and manufacturing processes would affect differently the GI, GL, InI and glycemic response of breads. We did not find differences in GI among different breads, only Wholemeal and Alfacar white breads had lower GI than glucose. GL and InI were lower after the intake of the breads than after the glucose. Only the Wholemeal had lower GL compared with the rest of the breads, except Alfacar, which was similar to Wholemeal and to the other white breads. On the other hand, the Wholemeal induced the highest release of GIP and PP, whereas no differences among the breads for appetite feelings were found. However, the amount of bread administered for the determination of GI (50 g of available carbohydrates) is a limitation for the evaluation of satiety and gastrointestinal hormone responses, since breads were not provided in isocaloric manner and particularly the wholemeal bread, provided an extra amount of energy that could affect these responses. Furthermore, only 50 g of available carbohydrates is a low amount of energy, far from a standard breakfast providing 25%–30% of daily energy. 

Fardet *et al.* [16] summarized glycemic responses of white and wholemeal breads, varying from 27 (barley bread with 75% whole grains) to 95 (extremely porous French baguette). Previous studies did not find significant differences among GI from a variety of typical French breads [17] and, contrary to our results, in that case, the wholemeal bread did not have the lowest GI value. Burton *et al.* [29] reviewed a number of factors influencing the glycemic response in carbohydrate foods, including the nature of monosaccharide components, the structure of the starch component and resistant starch, the degree of starch damage through food processing, food form and particle size, and the inclusion of whole kernels, viscous fibers and organic acids. In addition, interaction between starch and protein limits accessibility to α-amylases by total or partial starch encapsulation [16]. Mechanisms for reduction of the glycemic response appear to be changes in gastric emptying rate and starch amylolysis, involving starch gelatinization and retrogradation [29]. On the other hand, the Wholemeal had similar GL to the Alfacar and lower than the rest of the white breads. In this case, GLs were lower for all breads compared to glucose. The lower GL of the Wholemeal can be attributed to the presence of high amount of fiber but also to the lower amount of available carbohydrates in a 40 g serving (15 g for the wholemeal bread). The postprandial glucose response has been previously reported to be unaffected by the fiber content of the breads [30]. Those authors found that only the inclusion of different refined grains or bran, but not the milled whole-wheat flour, was able to decrease GI. Furthermore, the presence of intact structures not accessible to human amylases that may delay gastric emptying or create a barrier to starch digestion is apparently more effective than dietary fiber *per se* in improving glucose metabolism irrespective of the type of cereal [31]. In addition, it has been proposed that an increased density or compactness of the bread loaf led to significant reductions in glycemic response and glycemic index [32]. Therefore, the lower GL found in the Wholemeal bread that would be predictive of a lower glycemic response may be due not only to the presence of fiber and whole-grains but also to the lower amount of available carbohydrates, as well as to the compactness of the crumb that results in difficult starch digestion. The glycemic response of Alfacar white bread was also lower, in this case probably due to the inclusion of low baking capacity wheat flour that probably allow a low gelatinization and starch degradation and absorption [29]. Typical organic acids, such as those produced during fermentation of Alfacar bread, including acetic and lactic acid, were demonstrated to slow gastric emptying [16] and could contribute to modulate the glycemic response. Candeal-flour is a medium-soft wheat flour with low gluten content and short fermentation time that generates less organic acid, which might influence subsequent glycemic response [16]. Previous results have indicated that partially-baked frozen white wheat bread has lower starch digestibility than directly baked bread [33]. Starch digestibility is decreased by resistant starch generated during freezing [33]. In addition, partial-baking may cause a lower degree of starch granule swelling than does conventional baking, leading to a decreased glycemic response [34]. However, our results do not support that assumption since Ordinary and Precooked-frozen had similar glycemic response; therefore, more studies are needed to evaluate the glycemic response of different partially baked frozen breads.

The GI concept has been included in dietary guidelines given by health professionals, and it is increasing the number of marketing departments of food industry that are interested in low-GI products. There was a marked interest in the measurement of glycemic response and GI of foods by researchers to obtain after commercial application purposes. Nevertheless, it was indispensable to develop a standard glycemic index methodology to avoid variations among different authors. This lack of systematic procedure have been resulted in ambiguous results in scientific literature, and our results may be affected when we compared our results with others authors. Brouns *et al.* [21] defined the most important recommendations and we fulfilled most of them. 

Despite differences in glycemic responses for the Wholemeal bread, InI was similar for all breads but was significantly lower than after glucose intake. The effect of different types of bread on insulin secretion is controversial. In accordance with our results, Najjar *et al.* [35] reported no differences in postprandial insulin secretion after the intake of white, sourdough or whole-wheat breads and Mofidi *et al.* [36] described that generally, whole-grain breads did not have beneficial metabolic responses. In addition, other authors have found higher insulin levels after the intake of high-fiber rye bread [37] or lupin bread [38], while others have reported that the effect depends on the type of added fiber [39]. On the other hand, Rizkalla *et al.* [17] found a lower InI for the traditional baguette and bread made with leaven compared with other white and wholemeal breads. 

Besides controversy of the impact of GI on health, there is good or emerging evidence that low-GI diets reduce the risk for cardiovascular disease [40], stroke [41,42], and metabolic syndrome [43], among others [12]. All this suggests that GI influences physiological functions in a relevant way [12]. In addition, modulation of insulin secretion is very important to improve chronic diseases such as diabetes, obesity and cardiovascular diseases. However, there has been reluctance to incorporate GI into dietary recommendations since other authors have reported contradictory data [8,44]. More studies are needed to clarify this issue. A limitation of this study was the indirect estimation of available carbohydrate content of the bread samples that may underestimate available carbohydrates provided by each type of bread. Therefore, it is recommended to include resistant and retrograded starch analyses in future works. Both glycemic response and InI were lower after the intake of all breads compared with a glucose oral challenge providing the same amount of available carbohydrates. This fact suggests that 50 g of available CHO exerts a different effect if they are ingested alone or into a food matrix such as bread, and that other components present in the bread are influencing those responses [45]. However, GI is one determinant of glycemic response. Thus, eating a food as part of a mixed meal affects the glycemic response, but does not alter the food’s GI [12]. The degree to which the glycemic response of a food is altered in the presence of other foods depends on the amount and source (GI) of carbohydrate and the amounts and types of fat and protein added [45]. 

In relation to gastrointestinal hormones, there is no consensus regarding the effect of different breads. In the present work, we have only found that GIP and PP release were higher after intake of the Wholemeal compared with white breads, whereas there was no differential effect of any bread in ghrelin, GLP-1 and PYY. Previously, we have reported that a cereal-based bread enriched in fiber and proteins and with dried fruits decreased postprandial variation of ghrelin, GIP and GLP-1, while increased PP release [23], indicating that different components may modulate postprandial gastrointestinal hormones response. Hartvigsen *et al.* [39] reported no effect of adding arabinoxylan or β-glucan on ghrelin, GLP-1 and GIP secretion, but that only rye bread with kernels may decrease postprandial secretion of these incretins. Opposite to these results, Vitaglione *et al.* [46], evaluated a bread enriched in β-glucan and obtained a reduction on ghrelin (23%) and also reported higher PYY secretion (16%) compared to a control bread. Other authors have described no differential effect of white wheat or whole-grain breads on GIP and GLP-1 [35,36,37] and that only sprouted-grain bread induced a greater GLP-1 secretion compared with whole-grain or white wheat breads [36,37]. Furthermore, Weickert *et al.* [47] found a lower postprandial modification of ghrelin and PYY after the ingestion of wheat fiber, but not with oat fiber-enriched bread. Therefore, it seems that the type of cereals and ingredients included in manufacturing breads mainly influences gastrointestinal hormone release. When comparing glucose with breads, we found differences in the postprandial response of ghrelin, GLP-1, GIP and PP, but not PYY. To the best of our knowledge, only An *et al.* [48] have compared the effect of oral glucose load and steamed-bread challenge in type 2 diabetics, and they described that only PYY and ghrelin responded differently to both interventions. Therefore, more studies are needed to ascertain the effect of breads composition on gastrointestinal hormone releases.

We did not observe any differences in the appetite ratings after the intake of different white breads or in comparison with the Wholemeal bread. A number of works have reported a positive influence of dietary fiber on satiety and hunger [23,38,39,46,49,50,51,52,53,54], while others have reported no effect [47,55,56,57]. A limitation of this study is the low amount of calories provided by the bread, far away for recommended intakes. In addition, the wholemeal bread provided an extra amount of energy that could not be neglected. Another limitation when analyzing appetite and gastro-intestinal hormone response data is the protocol used, as those analyses require that foods be offered in an isocaloric manner. Therefore, more studies using standardized guidelines procedures [22] that compare well described breads are needed in this area. In addition, as previously reported [58], the sensory acceptance was more favorable to white breads than to Wholemeal bread in Spanish population, what might be due to the consistency of the bread. This can be another limitation of this study since palatability affect appetite ratings [59]. Despite that, we are aware that whole grain breads have better nutritional profile and recommend their consumption primarily instead of white breads. 

When analyzing correlations, we observed that glycemic response is related to satiety and fullness. However, small correlation coefficients indicate that the influence of GI, GL and InI on the appetite is low and, therefore, other, more powerful, factors must be implicated. According to our results and those of other authors, a higher satiety response has been observed after the ingestion of food with higher glycemic responses [60,61]. On the other hand, other work shows inverse relations [62]. However, as mentioned before, the use of portions of bread providing different amount of calories, makes it difficult to evaluate these results. In addition, here we report that PYY postprandial variation is correlated with the decrease of hunger, while we did not find any correlation between postprandial ghrelin, GLP-1, GIP or PP and satiety feelings or energy intake. These results may suggest that PYY is more implicated in the control of appetite feelings than the other gastrointestinal hormones. However, since appetite feelings are not regulated by a unique satiety hormone or any unique profile of hormones, it is difficult to explain the satiety effects through hormone variations [63]. Previously, we reported an indirect relation between postprandial ghrelin and appetite scores and direct association between ghrelin, GIP and PP with and *ad libitum* energy intake [23]. However, in the present work we found no relation between these parameters. Therefore, more studies are needed to establish the role of all gastrointestinal hormones in the control of food intake that may be influenced by the specific nourishment ingested. Finally, correlation between incretins, PP and PYY and glycemic and insulinemic responses confirms their contribution to carbohydrate metabolism.

## 6. Conclusions

In summary, this is the first study evaluating the glycemic response, insulin response, appetite and gastrointestinal hormone responses, following the intake of the five most common breads consumed in Spain differing in composition and manufacturing process. We did not find relevant differences in glycemic index or insulinemic index within the breads. We found differences in GL among the tested breads, mainly in the Wholemeal bread, and more interest focus on glycemic load should be addressed. We also did not obtain relevant differences in either appetite ratings or gastrointestinal hormone responses between breads. Despite this, more emphasis on encouraging the consumption of different breads, specially whole wheat breads, should be made by national authorities in order to promote healthier and varied diets, and therefore, to establish healthy lifestyles. 

## Supplementary Files

Supplementary File 1
