# Supplementary material for: Glycemic Responses, Appetite Ratings and Gastrointestinal Hormone Responses of Most Common Breads Consumed in Spain. A Randomized Control Trial in Healthy Humans
Source: Nutrients. 2015 May 27;7(6):4033–53. doi: 10.3390/nu7064033 (PMC4488771; doi:10.3390/nu7064033)
Supplement: Supplementary File 1 [file nutrients-07-04033-s001.docx]

Supplementary Materials

**Table S1.** Gastrointestinal hormones plasma concentrations (AUC) after the intake of five different breads consumed in Spain.

|  | **Ordinary** | **Precooked-Frozen** | **Candeal-Flour** | **Alfacar** | **Wholemeal** | **Glucose** |
| --- | --- | --- | --- | --- | --- | --- |
|  | Mean ± SEM | Mean ± SEM | Mean ± SEM | Mean ± SEM | Mean ± SEM | Mean ± SEM |
| Ghrelin (pg/mL·min) | −3844 ± 1098 | −3487 ± 780 | −4351 ± 798 | −4842 ± 737 | −4768 ± 1013 | −5338 ± 632 |
| GIP (pg/mL·min) | 5367 ± 480 ^a^ | 5272 ± 424 ^a^ | 6131 ± 463 ^ab^ | 6339 ± 510 ^ab^ | 6833 ± 578 ^b^ | 6072 ± 489 |
| GLP-1 (pg/mL·min) | 1459 ± 524 | 1675 ± 793 | 1492 ± 409 | 1758 ± 394 | 1418 ± 395 | 2202 ± 287 |
| PP (pg/mL·min) | 8338 ± 1606 ^ab*^ | 6385 ± 1269 ^a^ | 8272 ± 1576 ^ab*^ | 7940 ± 1236 ^ab*^ | 11,381 ± 2438 ^b*^ | 3659 ± 848 |
| PYY (pg/mL·min) | 1341 ± 1290 | 2275 ± 1686 | 1743 ± 1062 | 1813 ± 810 | 1592 ± 606 | 789 ± 199 |

Values are expressed as the mean ± SEM (*n* = 22). A negative value indicates a postprandial decrease (negative response). LMM was used to compare different breads with glucose adjusted by age and gender and using Sidack test for the marginal means. AUC, area under the curve; GIP, gastric inhibitory polypeptide; GLP-1, glucagon-like peptide-1; LMM: linear mixed-effects model; PP, pancreatic polypeptide; PYY, peptide YY; SEM: standard error of the mean; * indicates differences *versus* glucose. Different superscript letter indicates significant differences between breads; *p <* 0.05 was considered significant.

**Table S2.** Postprandial appetite ratings (AUC) energy intake the day before (24 h before), at the *ad libitum* lunch and the day of the intervention (24 h after), and sensory acceptance after the intake of five different breads consumed in Spain.

|  | **TESTED BREADS** | | | | |
| --- | --- | --- | --- | --- | --- |
|  | **Ordinary** | **Precooked-Frozen** | **Candeal-Flour** | **Alfacar** | **Wholemeal** |
|  | Mean ± SEM | Mean ± SEM | Mean ± SEM | Mean ± SEM | Mean ± SEM |
| Hunger (cm·min) | 6787 ± 822 | 6216 ± 815 | 6336 ± 903 | 6687 ± 887 | 7169 ± 904 |
| Satiety (cm·min) | 7220 ± 891 | 7022 ± 863 | 6836 ± 891 | 6631 ± 838 | 7693 ± 951 |
| Fullness (cm·min) | 7209 ± 943 | 7133 ± 878 | 7396 ± 858 | 6527 ± 853 | 7942 ± 938 |
| Prospective consumption (cm·min) | 6089 ± 743 | 5539 ± 746 | 6121 ± 804 | 6690 ± 806 | 7112 ± 806 |
| Composite appetite score (cm·min) | 6757 ± 846 | 6418 ± 790 | 6551 ± 848 | 6553 ± 836 | 7441 ± 875 |
| Desire to eat sweet (cm·min) | 6556 ± 1187 | 5999 ± 1276 | 5663 ± 1141 | 5335 ± 1190 | 5992 ± 1128 |
| Desire to eat salty (cm·min) | 3546 ± 904 | 4258 ± 965 | 3799 ± 769 | 5468 ± 980 | 5332 ± 909 |
| Desire to eat savoury (cm·min) | 3758 ± 864 | 3964 ± 957 | 4124 ± 938 | 4788 ± 874 | 4939 ± 914 |
| Desire to eat fatty (cm·min) | 1161 ± 379 | 1543 ± 529 | 1565 ± 495 | 1424 ± 398 | 2029 ± 621 |
| Intake 24 h before (kcal) ^†^ | 2174 ± 142 | 2909 ± 734 | 2173 ± 174 | 2138 ± 139 | 2171 ± 138 |
| *Ad libitum* lunch eaten (kcal) ^‡^ | 851 ± 41 | 811 ± 47 | 853 ± 55 | 808 ± 55 | 839 ± 47 |
| Intake 24 h after (kcal) ^§^ | 1987 ± 125 | 2014 ± 112 | 1893 ± 117 | 1884 ± 113 | 1977 ± 113 |
| Visual appearance | 12 ± 3 ^a^ | 12 ± 3 ^a^ | 13 ± 3 ^a^ | 22 ± 5 ^a^ | 43 ± 7 ^b^ |
| Bread smell | 19 ± 4 ^a^ | 22 ± 4 ^ab^ | 16 ± 4 ^a^ | 27 ± 5 ^ab^ | 35 ± 6 ^b^ |
| Bread aftertaste | 14 ± 3 ^a^ | 15 ± 4 ^a^ | 18 ± 4 ^a^ | 25 ± 4 ^a^ | 47 ± 7 ^b^ |
| Bread palatability | 13 ± 3 ^a^ | 16 ± 4 ^a^ | 19 ± 5 ^a^ | 24 ± 4 ^a^ | 58 ± 6 ^b^ |

Values are expressed as the mean ± SEM (*n* = 22). LMM was used to compare different breads adjusted by age and gender and using Sidack test for the marginal means. No significant differences (*p* < 0.05) were found. ^†^ Dietary intake 24 h before the day of the intervention (kcal). **^‡^** *Ad libitum* lunch meal intake 4 h after the intervention **^§^** Dietary intake 24 h after the intervention (kcal). AUC, area under the curve; LMM: linear
mixed-effects model; SEM: standard error of the mean; different superscript letter indicates significant differences between breads; *p <* 0.05 was considered significant.

© 2015 by the authors; licensee MDPI, Basel, Switzerland. This article is an open access article distributed under the terms and conditions of the Creative Commons Attribution license (http://creativecommons.org/licenses/by/4.0/).
